# Data describing the association between rs266729 polymorphism inadiponectin promoter gene and Type 2 Diabetes Mellitus

**DOI:** 10.1016/j.dib.2016.11.040

**Published:** 2016-11-21

**Authors:** Saiedeh Erfanian, Malihe Moradzadeh, Kavous Solhjoo, Abdolreza Sotoodeh Jahromi

**Affiliations:** a Research Center for Non-Communicable Diseases, Jahrom University of Medical Sciences, Jahrom, Iran; b Department of New Sciences and Technology, Mashhad University of Medical Sciences, Mashhad, Iran; c Zoonoses Research Center, Jahrom University of Medical Sciences, Jahrom, Iran

**Keywords:** Polymorphism, rs266729, Adiponectin gene and type 2 diabetes

## Abstract

This article investigates whether there is an association between the rs266729 polymorphism in adiponectin promoter gene with metabolic parameters and disease status in 300 type 2 diabetes patients and 300 healthy adults from Jahrom city, Iran. The variants (G/C) were tested by polymerase chain reaction-restriction fragment length polymorphism method (RFLP) and metabolic parameters (glucose, cholesterol, HDL and LDL cholesterol) were measured using biochemical methods. However, no differences were detected between the haplotypes investigated, and the data obtained from our lab shown association of the ADIPOQ promoter polymorphism neither with biochemical parameters, nor with disease status.

**Specifications Table**

**Table:** 

Subject area	*Biology*
More specific subject area	*Endocrinology, diabetes*
Type of data	*Table, image*
How data was acquired	*RFLP, biochemical assay*
Data format	*Raw, analyzed*
Experimental factors	*Genotyping of controls and diabetic Patient*
Experimental features	*Polymorphism rs266729 of ADIPOQ gene was tested by RFLP using* HhaI *enzyme and metabolic parameters were measured using biochemical methods in type 2 diabetic patients and healthy adults*
Data source location	*Jahrom university, Iran*
Data accessibility	*Data supplied with this article*

**Value of the data**

• We evaluated the association one of the adiponectin gene polymorphisms with diabetes type 2.
• This data is useful for characterising the relationship between heritable traits and the polymorphism.
• Our data can provide insight into methodology and considerations for investigation of polymorphisms in non-coding regions.

## 1 Data

We investigated the potential association between the ADIPOQ gene polymorphism and metabolic parameters in type 2 diabetic patients. The clinical data and baseline characteristics of the participants are shown in Table 1. The frequency distribution of genotypes (G/C) rs266729 polymorphism in healthy subjects and patients is given in Table 2. As GG mutant genotype frequencies between the two groups of diabetic patients and healthy, as well as the frequency of the G allele showed no significant difference between the groups (*P*≥0.05). The relationship between the compositions of the CC genotype against GC+GG with biochemical factors in both patients and controls were evaluated. As shown in Table 3. No significant relationship was observed between biochemical factors and this polymorphism (*P*≥0.05).

## 2 Experimental design, materials and methods

### 2.1 Characteristics of patients

A cross sectional study was conducted and samples were determined using statistical calculations. All participants were in the Fars area and Composition of the population in this area is rare. This research conforms to the declaration of Helsinki and approved by the ethics research committee of Jahrom University of Medical Sciences. Each participants had consent to partake in the study and, based on the testimonial, they could leave the study. This study was approved by the Research Ethics Committee of Jahrom University of Medical Sciences.

### 2.2 Biochemistry tests

People lipid profile (glucose, triglycerides, LDL and HDL) were measure enzymatically using biochemical kits (Pars Azmoon, Iran).

### 2.3 Genotyping

3–5 ml of venous blood was taken from each of the subjects after 12 h of fasting. DNA was extracted from nucleated blood cells, according to the DNA extraction protocol kit (cinagenne Co., Iran).

Sequence of ADIPOQ gene containing the polymorphism was amplified, using a PCR method with specific primers F: 5′_ GGTGGACTTGACTTTACTGG -3′ and R: 5′_ TAGAAGCAGCCTGGAGAA -3′ [1]. PCR reactions were performed in tubes 0.2 ml of PCR Premix (*Bioneer*, Daejeon, Korea). PCR program were as follows: 94 °C for 5 min, with 35 cycles of 94 °C for 30 s, 55 °C for 30 s, and 72 °C for one min and final extension 72 °C for 10 min. PCR products (10 μl) of ADIPOQ gene were digested in a 20-μl reaction volume for 24 with 1.5 U of HhaI at 37 °C (Fermentase Co, Germany). The digested PCR product was separated on 2% agarose gel and analyzed using G:Box transilluminator System (Syngene, Frederik,MD, USA). The HhaI product G allele yielded fragments of 212 and 122 bp; C allele, 334 bp (Fig. 1). Statistical analyses were carried out using commercial available software (SPSSv.20.0package, IBM, Chicago, IL).

## Appendix A Supplementary material

Supplementary Fig. 1 PCR-amplified of Adiponectin gene (334-bp).

.

Supplementary Fig. 2 The HhaI digestion profiles of product G allele yielded fragments Lane 2: GC genotype (heterozygous) Lane 3: GG genotype (homozygous, mutant type) and line 1 CC genotype.
