# Supplementary material for: Data describing the association between rs266729 polymorphism inadiponectin promoter gene and Type 2 Diabetes Mellitus
Source: Data Brief. 2016 Nov 21;9:1138–40. doi: 10.1016/j.dib.2016.11.040 (PMC5133470; doi:10.1016/j.dib.2016.11.040)
Supplement: Supplementary file 1 — Supplementary material [file mmc1.docx]

**Conflicts of Interest**

Manuscript title: Data describing the effect of rs17360539 polymorphism of the adiponectin promoter gene on diabetic patients

**Authors:** Saiedeh Erfanian^1^, Malihe Moradzadeh^2^, Kavous Solhjoo^3^**,** Abdolreza Sotoodeh Jahromi^1^

**Affiliations:**

1-Research Center for Non-Communicable Diseases, Jahrom University of Medical Sciences, Jahrom, Iran 2-Department of New Sciences and Technology, Mashhad University of Medical Sciences, Mashhad, Iran

3-Zoonoses Research Center, Jahrom University of Medical Sciences, Jahrom, Iran.

The authors declare that they have no competing interests.

**Conflicts of interest: none**
